# Cardiac CT in Large Vessel Occlusion Stroke for the Evaluation of Non-Thrombotic and Non-Atrial-Fibrillation-Related Embolic Causes

**DOI:** 10.3390/neurolint17020025

**Published:** 2025-02-07

**Authors:** Karim Mostafa, Cosima Wünsche, Sarah Krutmann, Carmen Wolf, Schekeb Aludin, Naomi Larsen, Alexander Seiler, Domagoj Schunk, Olav Jansen, Hatim Seoudy, Patrick Langguth

**Affiliations:** 1 Department of Radiology and Neuroradiology, University Medical Center Schleswig-Holstein, Campus Kiel, Arnold-Heller-Street 3, 24105 Kiel, Germany; cosima.wuensche2@uksh.de (C.W.); carmen.wolf@uksh.de (C.W.); schekeb.aludin@uksh.de (S.A.); naomi.larsen@uksh.de (N.L.); olav.jansen@uksh.de (O.J.); patrick.langguth@uksh.de (P.L.); 2 Department of Neurology, University Medical Center Schleswig-Holstein, Campus Kiel, 24105 Kiel, Germany; sarah.krutmann@uksh.de (S.K.); alexander.seiler@uksh.de (A.S.); 3 Advanced Clinician Scientist Programme, Faculty of Medicine, University of Kiel, 24105 Kiel, Germany; 4 Interdisciplinary Emergency Department, University Medical Center Schleswig-Holstein, Campus Kiel, 24105 Kiel, Germany; domagoj.schunk@uksh.de; 5 DZHK (German Centre for Cardiovascular Research), Partner Site Hamburg/Kiel/Lübeck, 24105 Kiel, Germany; hatim.seoudy@uksh.de; 6 Department of Internal Medicine III, Cardiology, Angiology and Critical Care, University Medical Center Schleswig-Holstein, 24105 Kiel, Germany

**Keywords:** cardiac CT, stroke etiology evaluation, cardioembolic stroke, large vessel occlusion stroke assessment

## Abstract

**Background:** The purpose of this study is the evaluation of imaging findings of acute-phase cardiac CT (cCT) in stroke patients with large vessel occlusion (LVO) to identify potential cardioembolic sources (CES) in patients without intracardiac thrombi and atrial fibrillation (AF). **Material and Methods:** This retrospective study included 315 patients with LVO who underwent cCT imaging in the acute stroke setting. The images were analysed for 15 imaging findings following the established minor and major cardioembolic risk factors. The final stroke aetiology was determined using the TOAST classification through interdisciplinary consensus following a thorough clinical evaluation. Multivariate regression analysis was performed to identify imaging findings associated with CES. **Results:** A cardioembolic aetiology was identified on cardiac CT in 211 cases (70%). After adjustment for AF and intracardiac thrombi, the multivariate regression analysis revealed significant associations with left ventricular dilation (adjusted odds-ratio (AOR) 32.4; 95% CI 3.0–349; *p* = 0.004), visible interatrial right-to-left shunt (AOR 30.8; 95% CI 2.7–341.3; *p* = 0.006), valve implants (AOR 24.5; 95% CI 2.2–270.9; *p* = 0.009), aortic arch atheroma grade > II (AOR 6.9; 95% CI 1.5–32.8; *p* = 0.015) and post-ischaemic myocardial scars (AOR 6.3, 95% CI 1.2–34.1; *p* = 0.032) as independent risk factors for a cardioembolic aetiology. The combined model achieved an area under the ROC curve of 0.83. **Conclusions:** In patients with LVO without AF and intracardiac thrombi as a cause, the presence of left ventricular dilatation, interatrial right-to-left shunts, valve implants, post-ischaemic myocardial scarring and advanced aortic arch atheroma (grade > 2) in particular is significantly associated with a cardioembolic cause of stroke and should be add-on evaluated in acute-phase cCT. Further investigations are warranted to confirm these associations.

## 1. Introduction

The accurate determination of the aetiology of ischemic stroke has a significant impact on its further treatment and secondary prevention strategies [1]. Despite extensive clinical investigation, approximately one third of all large vessel occlusion (LVO) strokes have no identifiable underlying pathogenic cause, resulting in a high number of cryptogenic strokes [2]. There are multiple potential origins of cryptogenic strokes, and this remains a topic of research, with studies suggesting that approximately 30% of all cryptogenic strokes are actually of cardiac origin—for example, due to undetected atrial fibrillation (AF)—while others suggest non-stenotic carotid plaques as the culprit lesions [1,2,3,4,5]. Regardless, patients with cardioembolic sources (CES) of stroke have the highest mortality rates and an increased risk of recurrent infarction compared to strokes of other aetiologies, making accurate and timely post-stroke evaluation critical [4,6,7]. Furthermore, given the overall populational ageing, with subsequently complex clinical situations in the framework of numerous severe comorbidities that can predispose patients to a large vessel occlusion stroke, the value of stroke aetiology evaluation is becoming increasingly important. However, the accurate determination of the cause of a stroke may become more difficult, especially in the face of the rising prevalence of AF and atherosclerotic disease in combination with the aforementioned factors [8].

In 1989, the WHO mentioned cardioaortic causes associated with cardioembolic stroke, which were subdivided into the generally recognised major and minor CES [1,9]. Here, transoesophageal echocardiography (TEE) is considered the gold standard for post-stroke cardiological imaging to identify most of these risk factors. However, TEE is a semi-invasive procedure that is often not feasible in the acute phase of stroke, potentially leading to the missed detection of CES. In recent years, cardiac computed tomography (cCT) has also become established as a non-invasive method of detecting CES and generally has the advantage of being able to be performed in the acute phase of stroke [5,10,11,12,13].

A thrombus identified on acute-phase cCT is almost definitively indicative of cardioembolic stroke in the absence of a competing cause [1,9]. Similarly, the presence of AF typically classifies a stroke as cardioembolic. However, when neither a thrombus nor AF is present, cCT is valuable in detecting other high- and low-risk cardioembolic factors [1]. This retrospective study aimed to assess the imaging findings for CES on cCT, based on major and minor cardioembolic factors, in patients with LVO without intracardiac thrombi and AF. 

## 2. Materials and Methods

This study was conducted in accordance with the tenets of the Declaration of Helsinki and its amendments and was approved by the local institutional review board of the Christian Albrecht University of Kiel (File No. D 524/48, date of approval: 29 July 2024).

### 2.1. Patient Selection and Data Collection

The inclusion criteria for this study were as follows: (1) diagnosis of intracranial LVO on CT angiography in the acute stroke setting; (2) availability of a cCT imaging study acquired in the acute stroke setting; (3) absence of an intracardiac thrombus on cCT imaging and absence of atrial fibrillation. Baseline demographic information and relevant clinical data for stroke classification were collected retrospectively by chart review as appropriate. 

### 2.2. Stroke and Cardiac CT Imaging Acquisition 

Stroke CT imaging was performed on two different CT systems (IQon and iCT; Philips Healthcare, Best, The Netherlands) in our tertiary stroke centre. Imaging was performed in two steps: (1) multimodal stroke CT imaging, including non-enhanced cranial CT, CT angiography of the intra- and extracranial vasculature up to the aortic arch and a CT perfusion study of the brain [10]; (2) ECG-guided cCT. The acquisition parameters for the cCT scan were as follows: 64 × 0.625 mm collimation, 0.27 s gantry rotation time, 100 kV or 120 kV tube voltage and 375 mA tube current.

In addition to the CT angiography and CT perfusion studies, 45 mL of contrast agent (Imeron, Bracco, Milan, Italy) was administered for the cCT, resulting in a total dose of contrast agent of 125 mL for the entire examination [10]. No additional medication, such as beta-blockers, was administered for the cCT.

The radiation dose for each examination was measured in patients using the standard dose indicator, the dose length product (DLP). The CT system determined the DLP for each scan and automatically recorded it in a dose report.

### 2.3. Imaging Analysis

**Figure 1:**
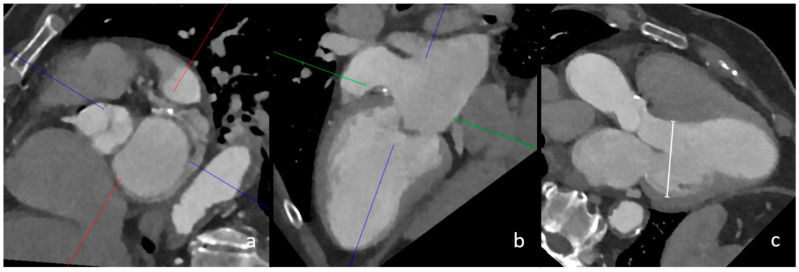
CT measurement of left ventricular dilatation in three-chamber view. In this image, the multiplanar reconstruction angulation of cCT imaging (**a**,**b**) is depicted. The diameter of the left ventricle in this female patient was 56 mm, which confirmed left ventricular dilatation (image (**c**), white line).

The cCT imaging was interpreted by two radiologists with several years of experience in cardiovascular imaging. The overall image and contrast quality was graded as (1) perfect, (2) good, (3) moderate or (4) non-diagnostic. Both radiologists assessed multiple cardiac imaging findings according to the recognised major and minor CES [1] (Supplementary Materials). In addition to the detection of atrial or ventricular thrombi, the presence of a left atrial septal pouch (LASP) and left atrial diverticulum (LAD), the configuration of the left atrial appendage (LAA; chicken wing, windsock, cauliflower and cactus), visual blood stasis in the LAA and the presence of interatrial contrast shunting as an indication of the presence of a persistent foramen ovale (PFO), the presence of valve implants, calcifications of the aortic and mitral valve, acute or previous myocardial infarction with or without ventricular aneurysms, cardiac tumours and the presence of left ventricular dilatation were assessed. The left ventricular diameters were approximatively measured in multiplanar reconstruction in a three-chamber view, as presented in Figure 1, following guidelines on chamber quantification, and a value of >53 mm in women and >59 mm in men was considered to indicate dilation [14]. Aortic arch atheroma was graded from 0 to 3, with grades 2 and 3 considered relevant (grade 1 = 0 to 4 mm plaque thickness, grade 2 ≥ 4 mm plaque thickness, grade 3 ≥ 4 mm plaque thickness with protrusion into the aorta; adapted from Tunick et al., 2000 and Amarenco et al., 1996 [15,16].

### 2.4. Stroke Classification

In the post-acute phase, all patients on the stroke unit underwent a comprehensive clinical and imaging assessment, including 24 h ECG monitoring and either transthoracic echocardiography (TTE) or transoesophageal echocardiography (TEE) given their availability. The presence of AF was assessed via the patient history, chart review and the results of the 24 h ECG monitoring. AF was considered absent when the patient history and findings of the ECG monitoring were inconspicuous. The extra- and intracranial vessels were examined in the acute stroke setting using CT angiography and, in selected cases, additionally with doppler ultrasound. The final stroke aetiology according to the TOAST classification was determined by interdisciplinary consensus based on all clinical and imaging findings, as well as the patient history. The strokes were categorised into one of five classes: (1) cardioembolism, (2) atherosclerosis of the large arteries, (3) small vessel disease, (4) stroke of other determined source, (5) embolic stroke of undetermined source (ESUS) [1]. ESUS was defined as a non-lacunar stroke without a history of or current AF, extra- or intracranial atherosclerosis causing luminal stenosis of more than 50% in the arteries supplying the ischaemic area and another significant CES or other specifically identified cause of stroke [2].

### 2.5. Statistical Analysis

A baseline demographic statistical analysis was performed as indicated. Multivariate logistic regression analysis was performed for the findings of cCT imaging to assess their influence on the outcome of a cardioembolic stroke aetiology. Based on these calculations, aggregated models of the imaging findings were created and tested with ROC analysis. Adjusted odds ratios and confidence intervals are reported as indicated.

## 3. Results

### 3.1. Characteristics of Study Population

This retrospective study included a total of 315 patients (60.6% female) who underwent additional cCT as a part of an acute LVO stroke assessment between 2018 and 2024. The mean age was 75.3 ± 14 years. The detailed demographic (e.g., age, gender) and clinical baseline characteristics are summarised in Table 1. The localisation of the vascular occlusion was the carotid artery (carotid-T) in 51 cases, the middle cerebral artery in 234 cases, the anterior cerebral artery in four cases, the basilar artery in eight cases, the posterior cerebral artery in 14 cases, the superior cerebellar artery in two cases and the posterior inferior cerebellar artery in one case. Known or newly diagnosed AF was present in 175 patients (55.5%).

### 3.2. Stroke Aetiology Classification

In the analysis of the stroke aetiology in the post-acute phase, a total of 221 LVO patients were classified as cardioembolic, 29 as large-artery atherosclerosis, 57 as ESUS and four as other sources. 

Of the 221 cardioembolic strokes, 174 patients had a known or newly diagnosed AF (78.7%), of which 24 patients also had a thrombus in the LAA. Among the patients without AF, there were eight cases with a thrombus in the LAA and 11 others with thrombi elsewhere in the heart or aorta. The thrombi outside the LAA were located at prosthetic valves (n = 2), in the left ventricle (n = 8) at post-ischaemic myocardial scars, in the ischaemia area in acute myocardial infarction (n = 1) and wall-adherent in the aortic arch (n = 1) (Table 1). Overall, AF and intracardiac thrombi accounted for 192/221 (86.9%) cardioembolic strokes.

### 3.3. Final Cohort Definition and Statistical Analysis

From the initial cohort of 315 patients, we identified a group of n = 121 patients with cardioembolic stroke and neither known nor newly diagnosed AF nor intracardiac thrombi. A flowchart showing the selection for the final cohort is given in the Supplementary Materials. The logistic regression analysis of this group revealed left ventricular dilatation (AOR 32.4, CI 3.0–349.0), the presence of interatrial right-to-left shunts (AOR 30.1; CI 2.7–341.3), aortic and mitral valve implants (AOR 24.5; CI 2.2–270.9), thrombotic aortic arch grade > 2 (AOR 6.9; CI 1.5–32.8) and a previous myocardial infarction (AOR 6.3; CI 1.2–34.1) as statistically significant imaging risk factors for a cardioembolic stroke (Table 2). In the aggregate model, the presence of these factors provided an area under the ROC curve of 0.83 for the discrimination of LVO strokes due to a cardioembolic aetiology from LVO strokes of other causes.

### 3.4. Additional Radiation Exposure for Acquisition of Cardiac CT

In our study, the median total dose length product (DLP) for the entire multimodal stroke and cCT examination was 1710 mGy*cm (IQR 1596–1816 mGy*cm), and, for the cardiac part (cCT) of the protocol, the DLP was 265 mGy*cm (IQR 219–344 mGy*cm).

## 4. Discussion

Up to 46% of all ischemic strokes are due to intracranial LVO [17]. Given their severity, these types of strokes are responsible for a disproportionate increase in morbidity and mortality compared to other types of stroke [17,18]. Most commonly, LVO stroke aetiologies are either cardioembolism or large artery atherosclerosis. The most common cause of cardioembolic stroke is AF [19,20,21]. In our study, a history or the presence of AF in combination with visible intracardiac thrombi could explain 86.9% of the cardioembolic strokes. These cases were excluded from our analysis to assess the relevance and frequency of other potential major and minor risk factors for cardioembolic stroke.

The main findings of this study are as follows. (1) In patients without AF or an intracardiac thrombus, whose LVO was classified as cardioembolic by interdisciplinary assessment, the following cCT findings were significantly associated with a cardioembolic aetiology: left ventricular dilatation, a visible interatrial right-to-left shunt, valve implants, a thrombotic aortic arch grade >II and an old post-ischemic myocardial scar (AUC 0.83). (2) The odds of classifying a stroke with LVO as cardioembolic were more than 20 times higher in the presence of left ventricular dilation, a visible right-to-left shunt and aortic or mitral valve implants, although the exact strength of this effect could not be determined. (3) In our cohort, AF and intracardiac thrombi were the most common cardioembolic causes of stroke with LVO.

### 4.1. Left Ventricular Dilatation

The strongest imaging risk factor among the known CES in our cohort was left ventricular dilatation measured in three-chamber view in multiplanar reconstruction (Figure 1). Left ventricular dilatation is a known risk factor for several cardiac diseases and has been associated with sudden cardiac death, among other complications [22]. Furthermore, geometric remodelling of the left ventricle has been shown to increase the risk of cardioembolic stroke and chronic heart failure [23]. In addition, left ventricular dilatation on cCT may indicate the presence of a dilated cardiomyopathy (DCM) [24]. In these patients, intraventricular congestion and thrombus formation are postulated to be promoted by reduced contractility and pump failure [25]. Furthermore, supraventricular and ventricular arrhythmias occur in up to 30% of patients with DCM, further increasing the thrombogenic potential [25]. In our exploratory analysis, the adjusted odds ratio of 32.4, with a remarkably wide confidence interval, suggests an association between left ventricular dilatation and a cardioembolic cause of stroke in patients with LVO; however, this needs to be further confirmed by studies with larger sample sizes.

### 4.2. Valvular Implants of the Mitral and Aortic Valve

Implants of the mitral valve and the aortic valve, which Adams et al. consider to be a major risk factor for embolism, were associated in our analysis with a 24.5-fold increase in the probability of a stroke being categorised as cardioembolic [1]. Here, the foreign material of the valve acts as an activating agent for thrombus formation. Furthermore, concomitant valve-related AF can further increase the risk of thrombus formation. In our cohort, three cases of mechanical aortic valves, one case of a bioprosthesis in the mitral valve and one case of a combined mechanical aortic valve and bioprosthetic mitral valve were reported. In two patients, the INR levels were outside the therapeutic range, while they were adequate for the rest of the patients. While the exact strength of the influence in our study can only be estimated, it is known that patients have an increased risk of stroke after valve replacement. Studies report up to 4% of strokes within the first 30 days after transcatheter aortic valve implantation (TAVI) and 4–9% after mitral valve replacement [26,27,28].

### 4.3. Visible Interatrial Shunt and PFO

**Figure 2:**
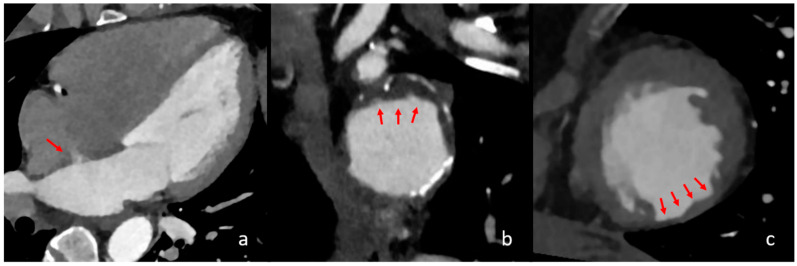
Different cardiac imaging findings with significant influence on a cardioembolic stroke aetiology. In the above images, different relevant cardiac findings are depicted that are associated with a cardioembolic stroke. In image (**a**), a four-chamber view is seen with contrast in the left atrium, left ventricle and aorta. The red arrow shows a persistent intra-atrial shunt, seen as contrast jet from the left to the right atrium. In image (**b**), reconstructed images of the aortic arch in the axial orientation show partially exulcerated plaques at the greater curvature of the aortic arch, marked with red arrows. In image (**c**), axial orientation slices of the left ventricle depict post-ischaemic myocardial scarring as the circumscribed thinning of the left ventricular myocardium, which is marked with red arrows.

The presence of visible interatrial shunting was associated with a 30.1-fold increase in the likelihood of a cardioembolic stroke (Figure 2). A PFO with a right-to-left shunt is conventionally considered a low-risk CES and is mainly discussed in the context of ESUS, where it is considered a high-risk source, especially in young patients [29]. Its prevalence in the general population is estimated at 18–34% [30,31]. While the reference standard for PFO detection is TEE, cCT allows for the depiction of the shunting effect of PFO, which is a relevant factor when considering its embolic potential, which has already been hypothesised [31,32,33]. However, there is contradictory evidence regarding our results, as Schiphorst et al. found echocardiographically relevant PFO not to be a relevant risk factor for stroke with LVO in young patients aged 18–65 years [34]. Our results suggest higher embolic potential for visible interatrial shunting in LVO, although our study included predominantly older patients. Further research is needed regarding the embolic potential in PFO and the comparison of CT and TEE considering the relevant shunting effect.

### 4.4. Aortic Arch Atheroma

Aortic arch atheroma graded as grade II or higher (>4 mm plaque thickness) was found to have a significant impact on cardioembolic stroke, with an adjusted odds ratio of 8.1. Although it is a common finding in CT imaging for stroke evaluation, it is not listed among the major or minor cardioembolic risk factors, even though different studies have associated it with these criteria (Figure 2) [15,35,36,37,38]. Our results are in good agreement with studies that have shown a 2.5- to 9-fold increased risk of stroke ofthrombotic aortic arch and a stroke recurrence risk of up to 11.9% per year for aortic arch atheroma thicker than 4 mm [15,37].

### 4.5. Previous Myocardial Infarction

Our study shows that the presence of a previous myocardial infarction and/or focal cardiac wall aneurysm, in the absence of AF and intracardiac thrombi, increases the probability of a stroke being classified as cardioembolic by a factor of 6.3. The relationship between myocardial infarction and stroke is well documented, as myocardial infarction is considered both a major and minor risk factor for cardioembolic stroke, depending on its severity [39,40,41]. The localised thinning of the myocardial tissue with reduced blood flow and/or aneurysmal dilatation in areas of a previous infarction can be easily visualised using cCT [42]. In addition, segmental myocardial hypoperfusion in the preserved myocardium on cCT indicates an acute myocardial infarction. In our study, such a finding was detected in one patient and was confirmed later. This also represents a potential risk for thrombus formation due to ischaemic myocardial hypokinesia (Figure 2) [43,44].

### 4.6. Additional Radiation Exposure and Contrast Agent for Acquisition of Cardiac CT

The additional radiation exposure and administration of contrast agents are disadvantages when a separate ECG-triggered cCT is integrated into the standard stroke imaging protocol. However, our results suggest that cCT imaging offers advantages in the early diagnosis of relevant CES. In contrast, the risk of additional radiation exposure is unlikely to influence the life expectancy, especially in an older patient group. Furthermore, additional chest CT imaging in the framework of acute stroke contributes little to the risk of fatal cancer, as recently reported by Lee et al. [45].

### 4.7. Clinical Implications

Cardiac CT imaging in acute ischaemic stroke is increasingly used as a first-line screening examination for CES in stroke, especially in the presence of an intracardiac thrombus [10,13,46,47]. When considering LVO stroke in the absence of an intracardiac thrombus, cCT allows the evaluation of most major and minor CES, but their presence alone cannot confirm cardioembolism [1]. According to the results of our study, we were able to show that the above-mentioned selected embolic factors have a significant association, with currently unclear strength, with cardioembolism as the final cause of stroke, which in turn was determined by comprehensive clinical assessment and interdisciplinary agreement. Therefore, we suggest the precise individual screening and stroke evaluation by cCT imaging for the presence of left ventricular dilatation, visible interatrial shunts, valve implants, thrombotic aortic arch grade > II and post-ischemic myocardial scars. In clinical practice, the presence of these findings should be discussed in an interdisciplinary setting and carefully considered in the context of a comprehensive clinical assessment when determining the aetiology of stroke, especially in cases of LVO stroke without a clear CES.

### 4.8. Limitations

This exploratory study has several limitations that need to be considered when interpreting the results. Firstly, it is necessary to mention the inherent selection bias given the population on which the results are based, as patients with LVO with neither AF nor intracardiac thrombi represent only a minority among stroke patients. Secondly, while significant associations between the factors were observed, the possibility of confounders affecting these relationships cannot be ruled out. Finally, although influences are hypothesised based on our data, the actual effect sizes of the factors of left ventricular dilatation, visible interatrial shunts and aortic and mitral valve implants remain unclear given the wide confidence intervals, which must be taken into account when interpreting our results. Thirdly, this study focused on cCT imaging findings only, without considering anticoagulation or antiplatelet management, which represents a limitation. Further studies with larger sample sizes are needed to comprehensively investigate the associations between the suggested imaging risk factors, anticoagulation or antiplatelet management and cardioembolic stroke.

## 5. Conclusions

In patients with acute ischaemic stroke due to LVO without evidence of AF and intracardiac thrombi and no competing aetiologies, cardiac CT imaging findings of left ventricular dilatation, visible interatrial shunting, aortic and mitral valve implants, post-ischaemic myocardial scarring and atheroma of the aortic arch > grade II may be significant risk factors for a cardioembolic stroke aetiology.

## Figures and Tables

**Table 1:** Study population and imaging findings.

	All Patients (n = 315)	No AF and No Thrombus Patients (n = 121)	*p*-Values
**Female, n (%)**	191 (60.3%)	64 (52.9%)	0.48
**Age, years**	75.3 ± 14.0	69.4 ± 16.1	<0.05
**Hypertension, n (%)**	197 (62.5%)	66 (54.5%)	0.48
**Diabetes, n (%)**	54 (17.1%)	18 (14.9%)	0.71
**Smoker, n (%)**	67 (21.3%)	28 (23.1%)	0.80
**Hyperlipidaemia, n (%)**	71 (22.5%)	25 (20.7%)	0.80
**CRP**	23.87 ± 40.25	21.65 ± 36.77	0.65
**HbA1c in %**	5.97 ± 0.85	5.89 ± 0.81	0.42
**TTE, n (%)**	184 (58.4%)	82 (67.8%)	0.40
**TEE, n (%)**	37 (11.7%)	26 (21.5%)	0.04
**Congestive heart failure, n (%)**	31 (9.8%)	9 (7.4%)	0.58
**AF, n (%)**	175 (55.5%)	0	<0.01
**Cardioembolic, n (%)**	221 (70.2%)	29 (24.0%)	<0.01
**Large-artery atherosclerosis, n (%)**	29 (9.2%)	28 (23.1%)	<0.01
**ESUS, n (%)**	57 (18.1%)	57 (47.1%)	<0.01
**Other source, n (%)**	8 (2.5%)	7 (5.7%)	0.14
**LASP, n (%)**	64 (20.3%)	17 (14.0%)	0.22
**LAD, n (%)**	86 (27.3%)	34 (28.1%)	0.91
**Thrombus, n (%)**	44 (13.9%)	0 (0.0%)	<0.05
**Visual stasis in LAA, n (%)**	120 (37.9%)	16 (13.2%)	<0.05
**Post ischaemic myocardial scar, n (%)**	44 (13.9%)	11 (9.1%)	0.26
**Aortic arch atheroma ≥ grade 2, n (%)**	31 (9.8%)	13 (10.7%)	0.86
**Visible interatrial shunt, n (%)**	11 (3.5%)	4 (3.3%)	1
**Cardiac tumor, n (%)**	3 (0.9%)	0 (0.0%)	0.56
**Left ventricular dilatation, n (%)**	21 (6.6%)	5 (4.1%)	0.5
**Aortic or mitral valve implants, n (%)**	22 (7.0%)	5 (4.1%)	0.04

CRP: C-reactive protein; TTE: transthoracic echocardiography; TEE: transoesophageal echocardiography; AF: atrial fibrillation; ESUS: embolic stroke of unclear source; LASP: left atrial septal pouch; LAD: left atrial diverticulum; LAA: left atrial appendage.

**Table 2:** Cardioembolic factors significantly associated with a cardioembolic stroke aetiology.

Factor	Adjusted Odds Ratio (AOR)	95% CI	*p*-Value
**Left ventricular dilatation**	**32.4**	**3.0–349.0**	**0.004**
**Intra-atrial shunting**	**30.1**	**2.7–341.3**	**0.006**
**Aortic or mitral valve implant**	**24.5**	**2.2–270.9**	**0.009**
**Thrombotic aortic arch grade II/III**	**6.9**	**1.5–32.8**	**0.015**
**Old myocardial infarction**	**6.3**	**1.2–34.1**	**0.032**

## Data Availability

Any data processed in this manuscript can be shared upon reasonable request.

## Supplementary Materials

The following supporting information can be downloaded at: https://www.mdpi.com/article/10.3390/neurolint17020025/s1, Figure S1: Implants of the mitral (a) and aortic valve (b); Figure S2: Patient selection flowchart; Table S1: High-risk and medium-risk sources for cardioembolic stroke [1].
