# Supplementary material for: Cardiac CT in Large Vessel Occlusion Stroke for the Evaluation of Non-Thrombotic and Non-Atrial-Fibrillation-Related Embolic Causes
Source: Neurol Int. 2025 Feb 7;17(2):25. doi: 10.3390/neurolint17020025 (PMC11858386; doi:10.3390/neurolint17020025)
Supplement: Supplementary file 1 [file neurolint-17-00025-s001.zip › neurolint-3451465-supplementary.pdf]

**Mechanical prosthetic valve**	**Mitral valve prolapse**
**Mitral stenosis with atrial fibrillation**	**Mitral annulus calcification**
**Atrial fibrillation (other than lone AF)**	**Mitral stenosis without atrial fibrillation**
**Left atrial/atrial appendage thrombus**	**Left atrial turbulence (smoke)**
**Sick sinus syndrome**	**Atrial septal aneurysm**
**Recent myocardial infarction (<4 weeks)**	**Myocardial infarction (>4 weeks, <6 months)**
**Left ventricular thrombus**	**Atrial flutter**
**Dilated cardiomyopathy**	**Lone atrial fibrillation**
**Akinetic left ventricular segment**	**Bioprosthetic cardiac valve**
**Atrial myxoma**	**Nonbacterial thrombotic endocarditis**
**Infective endocarditis**	**Congestive heart failure**
	**Hypokinetic left ventricular segment**
	**Patent foramen ovale**

Table S1. High-risk and medium-risk sources for cardioembolic stroke [1]
.

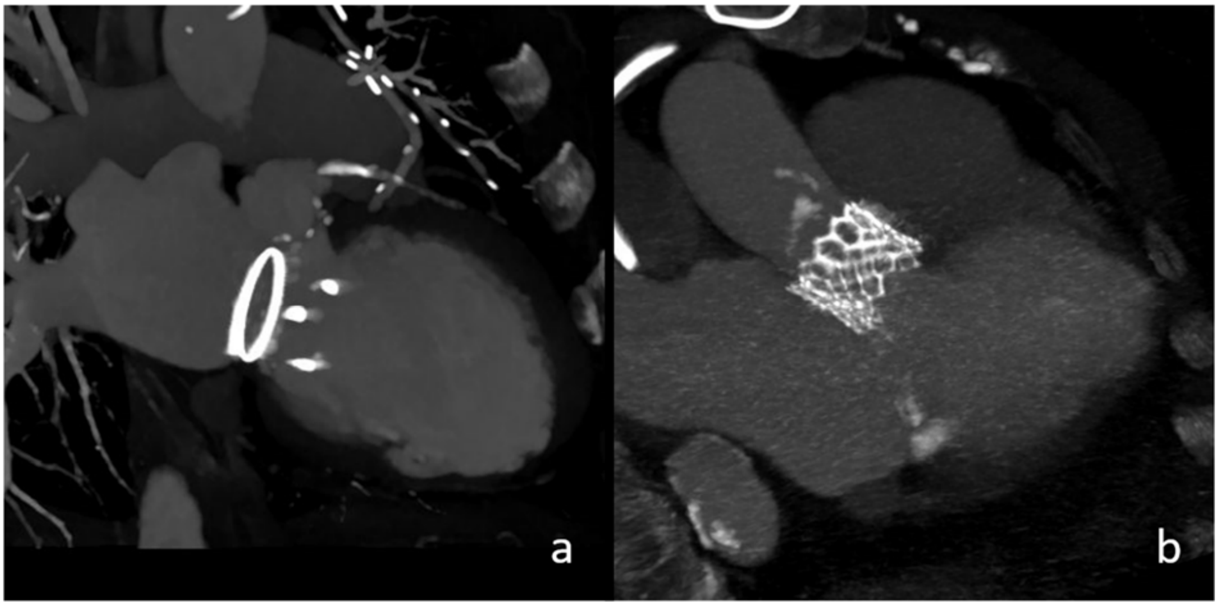

Figure S1. Implants of the mitral (a) and aortic valve (b).

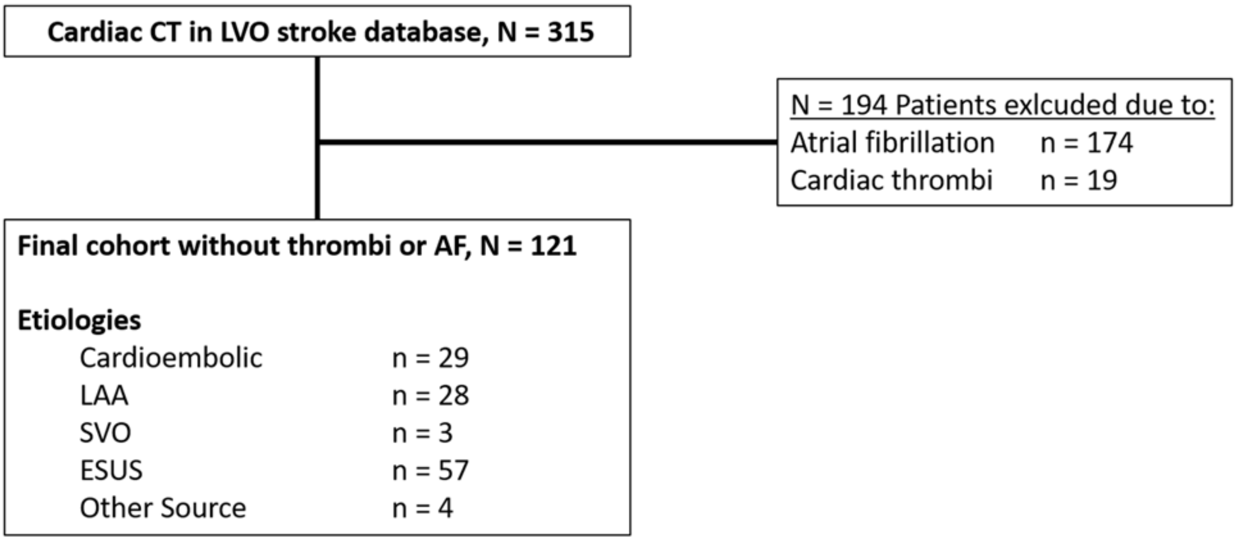

Figure S2. Patient selection flowchart.
